# Long-term cardiac outcomes of patients with HER2-positive breast cancer treated in the adjuvant lapatinib and/or trastuzumab Treatment Optimization Trial

**DOI:** 10.1038/s41416-020-0786-x

**Published:** 2020-03-16

**Authors:** Daniel Eiger, Noam F. Pondé, Dominique Agbor-Tarh, Alvaro Moreno-Aspitia, Martine Piccart, Florentine S. Hilbers, Olena Werner, Saranya Chumsri, Amylou Dueck, Judith R. Kroep, Henry Gomez, István Láng, Richard J. Rodeheffer, Michael S. Ewer, Thomas Suter, Evandro de Azambuja

**Affiliations:** 1 Institut Jules Bordet Institute and L’Université Libre de Bruxelles (U.L.B.), Brussels, Belgium; 2 AC Camargo Cancer Center, São Paulo, Brasil; 3 Frontier Science, Kingussie, United Kingdom; 4 Mayo Clinic, Jacksonville, FL USA; 5 Breast International Group (BIG), Brussels, Belgium; 6 Novartis Pharma AG, Basel, Switzerland; 7 Mayo Clinic, Jacksonville, FL USA; 8 Alliance Statistics and Data Center, Mayo Clinic, Scottsdale, AZ, USA; 9 Department of Medical Oncology, Leiden University Medical Center, Leiden, The Netherlands; 10 Instituto Nacional de Enfermedades Neoplasicas, Lima, Peru; 11 Istenhegyi Géndiagnosztika Private Health Center, Oncology Clinic, Budapest, Hungary; 12 Cardiovascular Department, Mayo Clinic, Rochester, MN USA; 13 MD Anderson Cancer Center, Houston, TX USA; 14 Department of Cardiology, lnselspital, Bern University Hospital, University of Bern, Bern, Switzerland

**Keywords:** Breast cancer, Targeted therapies

## Abstract

**Background:**

Cardiotoxicity is the most significant adverse event associated with trastuzumab (T), the main component of HER2-positive breast cancer (BC) treatment. Less is known about the cardiotoxicity of dual HER2 blockade with T plus lapatinib (L), although this regimen is used in the metastatic setting.

**Methods:**

This is a sub-analysis of the ALTTO trial comparing adjuvant treatment options for patients with early HER2-positive BC. Patients randomised to either T or concomitant T + L were eligible. Cardiac events (CEs) rates were compared according to treatment arm.

**Results:**

With 6.9 years of median follow-up (FU) and 4190 patients, CE were observed in 363 (8.6%): 166 (7.9%) of patient in T + L arm vs. 197 (9.3%) in T arm (OR = 0.85 [95% CI, 0.68–1.05]). During anti-HER2 treatment 270 CE (6.4%) occurred while 93 (2.2%) were during FU (median time to onset = 6.6 months [IQR = 3.4–11.7]). While 265 CEs were asymptomatic (73%), 94 were symptomatic (26%) and four were cardiac deaths (1%). Recovery was observed in 301 cases (83.8%). Identified cardiac risk factors were: baseline LVEF < 55% (vs > 64%, OR 3.1 [95% CI 1.54–6.25]), diabetes mellitus (OR 1.85 [95% CI 1.25–2.75]), BMI > 30 kg/m^2^ (vs < 25 mg/kg^2^, OR 2.21 [95% CI 1.40–3.49]), cumulative dose of doxorubicin ≥240 mg/m^2^ (OR 1.36 [95% CI 1.01–1.82]) and of epirubicin≥ 480 mg/m^2^ (OR 2.33 [95% CI 1.55–3.51]).

**Conclusions:**

Dual HER2 blockade with T + L is a safe regimen from a cardiac perspective, but cardiac-focused history for proper patient selection is crucial.

**Trial registration number:**

ClinicalTrials.gov Identifier: NCT00490139 (registration date: 22/06/2007); EudraCT Number: 2006–000562–36 (registration date: 04/05/2007); Sponsor Protocol Number: BIG2–06 /EGF106708/N063D.

## Background

Treatment with trastuzumab in Human Epidermal Growth Factor Receptor 2 (HER2)-positive breast cancer (BC) has dramatically changed the prognosis of early disease, reducing the risk of recurrence by 40% and risk of death by 34%.^1^ Clinically, the most significant adverse reaction to anti-HER2 treatment is cardiotoxicity, which is manifested most often as asymptomatic reduction in left ventricular ejection fraction (LVEF), though a minor subset of patients experience overt congestive heart failure (CHF) and, very rarely, cardiac death.^2^

Currently, dual HER2 blockade combining trastuzumab/pertuzumab (early and advanced setting) and trastuzumab/lapatinib (advanced setting) are part of the standard of care.^3–7^ Lapatinib (L), a reversible tyrosine kinase inhibitor (TKI) of HER1 and 2, is approved for the treatment of HER2-positive, trastuzumab-resistant metastatic BC in combination with trastuzumab (T), capecitabine or endocrine therapy.^7–9^ Little is known, however, about the potential of dual HER2 blockade with T + L for causing cardiotoxicity.^7,9^ Therefore, the objective of this cardiac-focused sub-analysis of the Adjuvant Lapatinib and/or Trastuzumab Treatment Optimization Trial (ALTTO Cardiac) is to provide a detailed picture of cardiotoxicity associated with the combination of T + L as well as further data on T-associated cardiotoxicity.

## Methods

### Study design

The Adjuvant Lapatinib and/or Trastuzumab Treatment Optimization ALTTO (Breast International Group [BIG] 2–06/EGF106708 and North Central Cancer Treatment Group [Alliance] N063D; clinicaltrials.gov identifier: NCT00490139) was an international multi-centre, open-label, phase 3 randomised clinical trial in patients with HER2-positive early BC, which has previously been reported.^5^ Briefly, 8381 patients with early HER2-positive BC were randomised to four different treatment arms, each given for one year: (1) T monotherapy; (2) L monotherapy; (3) sequential treatment with both drugs (T→L); (4) the combination of the two drugs (T+L). Anti-HER2 treatment could be given at the completion of all chemotherapy or concomitantly with taxanes, after the anthracycline component was finished (Fig. S1 in the appendix).

Patients had to have a baseline LVEF ≥ 50% measured by either echocardiography (ECHO) or multiple-gated acquisition (MUGA) scan, prior to anti-HER2 therapy. Patients treated with an anthracycline-based chemotherapy were eligible if cumulative doses of doxorubicin were ≤360 mg/m^2^ or epirubicin ≤ 720 mg/m^2^. Those with serious cardiac illness were not eligible. Further details regarding patients‘ selection and study design are available elsewhere.^5^

The ALTTO Cardiac population consists of patients randomised to T (2097 patients) and T + L (2093 patients) arms, as T→L and L treatments are not used in current clinical practice. A consort diagram is provided in Fig. S2.

### Cardiac monitoring

LVEF was assessed consistently with the same method (ECHO or MUGA) at baseline, weeks 13, 25, 37 and 52, and during follow-up on months 18, 24, 36, 48 and 60. After this period, LVEF was assessed yearly until year 10. Importantly, after a protocol amendment in March 2016, LVEF assessment after 5 years of follow-up was no longer mandatory.

In case of development of clinical signs or symptoms compatible with congestive CHF, unscheduled LVEF assessment and electrocardiogram (ECG) were performed. Furthermore, the following treatment interruption and discontinuation recommendations for anti-HER2 treatment were provided in the study protocol, as described below:

1. Patients who developed severe CHF, New York Heart Association (NYHA) class III or IV had T and or L permanently discontinued;
2. Patients who developed asymptomatic or mildly symptomatic CHF, NYHA class I or II had treatment temporarily interrupted and where managed according to the algorithm provided in Fig. S3 of the appendix, which included the possibility of re-introduction of anti-HER2 therapy.

### Cardiac endpoints

Cardiac events (CEs) were defined for the purpose of this analysis as follows. It is important to note that they are slightly different from the original ALTTO protocol definitions (Supplementary Table S1).

#### Asymptomatic CE

Asymptomatic significant LVEF drop, defined as an absolute decline of at least 10 percentage points from baseline and to below 50%, either by MUGA or ECHO. This event had to be confirmed with a 2nd LVEF assessment after 3 weeks of the 1st significant LVEF drop.

#### Symptomatic CE

NYHA class II, III or IV HF associated with a significant LVEF drop.

Symptomatic CE NYHA class II had to be confirmed with a 2nd LVEF assessment after 3 weeks of the 1st significant LVEF drop.

#### Cardiac death

Death definitely due to CHF, myocardial infarction or documented arrhythmia, or probable cardiac death within 24 h of a CE.

The following outcomes were further assessed, according to definitions below:

#### Acute recovery from CE

Two or more consecutive LVEF assessments of 50% or greater after a CE, irrespective of (cardiovascular) treatment. The date of the first LVEF assessment showing an increase of LVEF above 50% was considered the recovery date.

#### Occurrence of LVEF drop to less than 50% following acute recovery

After a patient reaches criteria for acute recovery from a CE, this event is defined as a 2nd LVEF drop to <50%, regardless of anti-HER2 treatment re-exposure or association with symptoms. Importantly, this outcome definition precludes capturing more than one CE per patient.

#### Time to development of CE

Time elapsed between the start of anti-HER2 treatment and the date of occurrence of a CE.

#### Time to recovery from CE

Time elapsed between the start of a CE and the recovery date.

#### Anti-HER2 treatment completion

Defined as completion of the pre-planned 52 weeks of anti-HER2 treatment.

#### Reasons for anti-HER2 non-completion

Defined as safety reason, recurrence of disease or other reasons (defined as a reason qualified as none of the two previous). Safety reason was further divided into cardiac safety (defined as permanent discontinuation due to a CE) and other safety (defined as permanent discontinuation due to an adverse event [AE]).

### Statistical analysis

Sample size calculation and statistical assumptions were previously reported.^5^

Data were extracted from the second analysis dataset, locked in Q12017. Baseline characteristics are described by treatment arms (T; T + L). The distribution of CEs, the median time for development of CEs, the incidence of recovery from CEs and the incidence of permanent treatment discontinuations are described by treatment arms (T, T + L). Categorical data were cross tabulated to generate proportion, then Chi-square tests to assess the stability of patients’ characteristics across treatment arms (T; T + L) were performed.

The reverse Kaplan-Meier method was used to determine the median follow-up (FU) time for the entire cohort and by treatment arms.

The primary endpoint was the incidence of CEs, defined as the sum of asymptomatic CEs, symptomatic CEs and cardiac deaths in the entire population and by treatment arms (T; T + L). Secondary endpoints include: (1) the incidence of asymptomatic CEs, symptomatic CEs and cardiac deaths in the entire population and by treatment arms, (2) risk factors for the occurrence of any CEs (cardiac risk factors), (3) the time to development of CEs, (4) the recovery rate, (5) the association between cardiac risk factors and absence of acute recovery following a CE, (6) the time to recovery after CEs and (7) mean LVEF at screening and during anti-HER2 treatment by treatment arms, (8) anti-HER2 treatment completion rates, and (9) reasons for anti-HER2 treatment non-completion.

Logistic regressions were fitted to assess the odds of CEs, with anti-HER2 randomised arms as the only predictor for the univariate analysis; other covariates were included in the multivariate analysis (baseline LVEF categories, presence of diabetes mellitus, body mass index categories and cumulative dose of doxorubicin or epirubicin). The logistic regression was repeated for each risk factor. Odd ratios and 95% CI were reported for both univariate and multivariate analyses, with a *p*-value less than 0.05 considered statistically significant.

The incidence of CEs over time by anti-HER2 randomised arms and the number of risk factors was assessed using the cumulative incidence plot of CEs over time, based on competing risks. Time was considered from randomisation.

Mean LVEF over time and 95% CI, by anti-HER2 randomised arms were plotted for visual inspection.

All analyses were performed using SAS version 9.4.

## Results

### Patients’ characteristics

With a median follow-up of 6.9 years (interquartile range [IQR] of 6.0–7.1), 2097 patients in T arm and 2093 patients in T + L arms were included in this sub-analysis. Baseline characteristics were well-distributed between treatment arms, except for diabetes mellitus (DM), with slightly more diabetic patients in the T arm (*p* = 0.024). Of note, most patients were younger than 65 years old (90% in both arms), few patients had any co-morbidity (28% in T arm and 27% in T + L arm), and a minority had a baseline LVEF between 50 and 54% (5% in both arms). ECHO was used for LVEF evaluation in 76% of patients in T arm and in 75% in T + L arm, while in the remaining cases MUGA was used. Most patients had received an anthracycline-based chemotherapy regimen (95%). Table 1 summarises baseline patients‘ characteristics according to treatment arm, focusing on cardiac-related features; Table S2 in the appendix includes all baseline characteristics.

**Table 1:** ALTTO Cardiac population.

Characteristic	T arm (2097 patients) *N* (%)	T + L arm (2093 patients) *N* (%)	*p*-value
Age (years)			0.918
<65	1881 (90)	1879 (90)	
≥65	216 (10)	214 (10)	
Baseline LVEF			0.765
50–54%	98 (5)	102 (5)	
55–64%	1065 (51)	1053 (50)	
>64%	934 (44)	937 (45)	
Missing	0	1 (<1)	
LVEF evaluation method			0.616
ECHO	1588 (76)	1571 (75)	
MUGA scan	509 (24)	522 (25)	
Any co-morbidity?			0.470
Yes	588 (28)	566 (27)	
No	1509 (72)	1527 (73)	
BMI (kg/m^2^)			0.916
<25	999 (48)	989 (47)	
25–30	679 (32)	675 (32)	
>30	419 (20)	429 (21)	
Hypertension			0.293
Yes	471 (22)	442 (21)	
No	1626 (78)	1651 (79)	
Diabetes Mellitus			**0.024**
Yes	128 (6)	95 (5)	
No	1969 (94)	1998 (95)	
Hypercholesterolemia			0.290
Yes	179 (9)	160 (8)	
No	1918 (91)	1933 (92)	
Radiotherapy laterality^a^			0.165
Left	745 (50)	790 (53)	
Right	736 (50)	698 (47)	
Bilateral	5 (<1)	2 (<1)	
Chemotherapy regimen			0.902
Anthracycline followed by taxane	1985 (95)	1983 (95)	
Non-Anthracycline (docetaxel + carboplatin)	112 (5)	110 (5)	
Median doxorubicin cumulative dose	237.62 mg/m^2^	237.84 mg/m^2^	–
Median epirubicin cumulative dose	350.86 mg/m^2^	349.75 mg/m^2^	–
Median follow-up (IQR) in years	6.9 (6.0–7.1)	6.9 (6.0–7.1)	–

*BMI* body mass index, *ECHO* echocardiogram, *LVEF* left ventricular ejection fraction, *IQR* interquartile range, *MUGA* multiple-gated acquisition, *T* trastuzumab, *T* *+* *L* trastuzumab + lapatinib.

^a^Percentages derived using 1486 patients on T arm and 1490 patients on T + L arm that received radiotherapy as denominator.

### Anti-HER2 treatment completion

Trastuzumab was discontinued before completion of 1 year of adjuvant treatment in 344 (16%) and 385 (18%) patients in T arm and T+L arm, respectively. Of those, reasons in T arm for discontinuation were: disease recurrence (15%), safety (36%) and other reasons (49%), whereas in T + L arm, trastuzumab discontinuation reasons were disease recurrence (7%), safety (35%) and other reasons (58%).

Lapatinib was discontinued by 674 (32%) of patients in T + L arm. Of those, reasons for discontinuation were: disease recurrence (3%), other reasons (37%) and safety (60%). Of note, only 74 (11%) patients discontinued lapatinib due to cardiac safety, while 164 (22%) patients receiving trastuzumab had to discontinue it. A summary of these findings is reported in Table S3.

### Cardiac events and mean LVEF

**Fig. 1:**
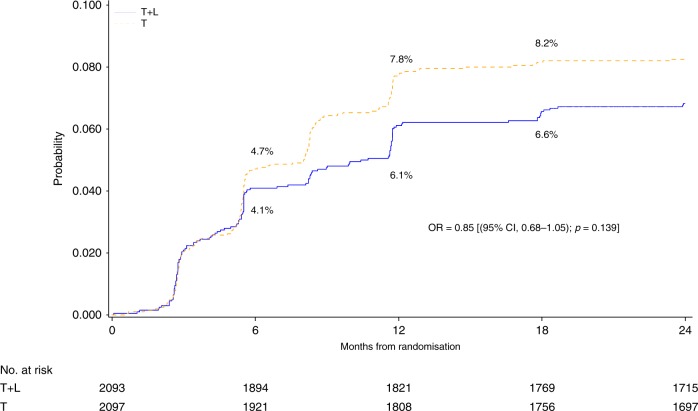
Cumulative incidence of CEs over time, per arm of treatment. There is a non-statistical difference of −1.4% in the rate of CE on T + L arm (7.9%) vs T arm (9.3%), with a multivariate OR of 0.85 [(95% CI, 0.68–1.05); *p* = 0.139]. The rates of CEs at the 6th month are 4.1% and 4.7% on T + L arm and T arm, respectively, increasing to 6.1% and 7.8% at the 12th month, and to a lesser extent to 6.6% and 8.2% at the 18th month, respectively.

Following the ALTTO first report, an additional set of 103 CEs have occurred, for a total of 363 CEs in 4190 patients, which represents an 8.7% rate of CEs. One-hundred and sixty-six (7.9%) patients in T + L arm versus 197 (9.4%) patients in T arm experienced a CE, a non-significant absolute difference of −1.4% (OR of 0.85; 95% CI, 0.68–1.05; *p* = 0.139). Two-hundred and seventy CEs (6.4%) occurred during anti-HER2 treatment and only 93 (2.2%) during follow-up (FU), with a median time to onset of 6.6 months (interquartile range: 3.4–11.7 months). The cumulative incidence curves of CEs over time in both T + L and T arms seem to be the steepest from randomisation to the 6th month than from the 6th to the 12th month of therapy and thereafter, as depicted in Fig. 1.

Asymptomatic CEs were the most frequent CE, with 265 events (6.3%), followed by 94 symptomatic events (2.2%) and four cardiac deaths (<0.1%), with an overall similar pattern of CE type according to treatment arm, as displayed in Table 2.

**Table 2:** Summary of cardiac events.

CEs, subtype and timing	All pts (4190) *N* (%)	T arm (2097) *N* (%)	T + L arm (2093) *N* (%)
Cardiac events	363 (8.6)	197 (9.3)	166 (7.9)
CE during anti-HER2 therapy	270 (6.4)	153 (7.2)	117 (5.6)
CE during follow-up phase	93 (2.2)	44 (2.1)	49 (2.3)
Asymptomatic CE	265 (6.3)	155 (7.4)	110 (5.3)
Symptomatic CE	94 (2.2)	40 (1.9)	54 (2.6)
Cardiac deaths	4 (<0.1)	2 (<0.1)	2 (<0.1)
Median time in months to develop a CE (range)	6.6 (3.4–11.7)	6.4 (3.6–11.7)	7.1 (2.9–16.6)

**Fig. 2:**
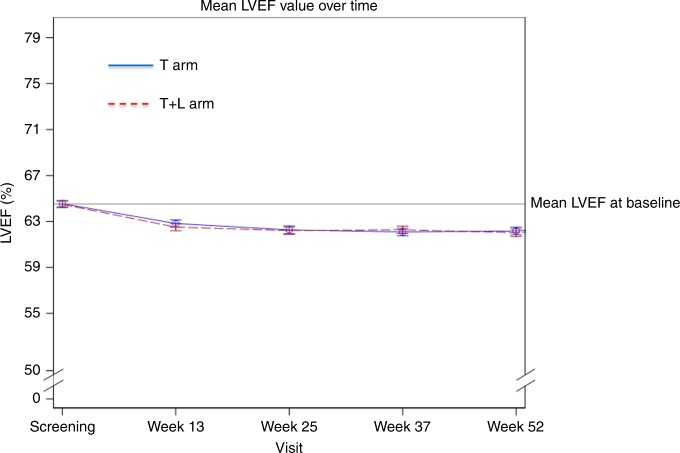
Mean left ventricular ejection fraction (mLVEF) over time according to treatment arm. After a minor decrement from screening to week 13 (T + L arm: 64.5–62.5%, T arm: 64.5–62.8%, respectively), mLVEF becomes stable in both treatment arms until the end of treatment (T + L arm: 62.0%, T arm: 62.2%).

Figure 2 shows a similar yet small decrease of cardiac function in both arms, from screening to week 13 after randomisation, which stabilises thereafter until the end of treatment.

### Cardiac recovery and second LVEF drop

Excluding two cardiac deaths in each treatment arm, 163 (83.6%) patients recovered from a CE in T arm and 138 (84.1%) recovered in T + L arm, with a median (range) time to recovery of 3.3 (0.0–79.0) and 3.5 (0.1–23.9) months, respectively. Anti-HER2 treatment re-exposure was done in 58 and 32 patients in T and T + L arms, respectively, following acute recovery. In these patients, a second LVEF drop to <50% was experienced by 15 patients (25.9%) in T arm and 12 patients (37.5%) in T + L arm.

Similar recovery rates were found amongst treatment arms according to type of CE (data summarised in Table S4).

### Cardiac Risk Factors and incidence of CEs according to cumulative number of risk factors

The following baseline characteristics were positively associated with CEs (hereafter cardiac risk factors) for the entire population, after adjusting for confounding variable in the multivariate analysis: pre anti-HER2 treatment LVEF <55% (vs >64%, OR = 3.1; *p* = 0.002), diabetes mellitus (OR = 1.85; *p* = 0.002), BMI > 30 kg/m^2^ (vs <25 mg/kg^2^, OR = 2.21; *p* < 0.001), cumulative dose of doxorubicin ≥ 240 mg/m^2^ (OR = 1.36; *p* = 0.039) and cumulative dose of epirubicin ≥480 mg/m^2^ (OR = 2.33; *p* < 0.001) (Table 3). No impact on CEs incidence was demonstrated for age ≥65 years (*p* = 0.064), hypercholesterolemia (*p* = 0.629), hypertension (*p* = 0.402), radiotherapy (*p* = 0.709), left side radiotherapy (*p* = 0.509) and dual HER2 blockade (*p* = 0.139) (Table S5). An ad-hoc analysis showed lower odds of experiencing a CE with T + L compared to T during the anti-HER2 treatment period (OR = 0.77; *p* = 0.038), but not during the FU period (OR = 1.14; *p* = 0.525); (Table S5).

**Table 3:** Cardiac risk factors.

Baseline characteristic	Cardiac events (%)	*N*	Univariate OR (95% CI)	Univariate *P*-value	Multivariate OR (95% CI)	Multivariate *P*-value
Baseline LVEF	363 (8.67)	4189				
>64%	102 (4.82)	2118	–	–	–	–
55–64%	225 (12.03)	1871	2.70 (2.12–3.44)	<0.001	2.32 (1.61–3.35)	<0.001
<55%	36 (18.00)	200	4.34 (2.87–6.55)	<0.001	3.10 (1.54–6.25)	0.002
Diabetes Mellitus	363 (8.66)	4190				
No	330 (8.32)	3967	–	–	–	–
Yes	33 (14.80)	223	1.91 (1.30 to 2.82)	<0.001	1.85 (1.25 to 2.75)	0.002
Doxorubicin cumulative dose	205 (10.90)	1880				
<240 mg/m^2^	109 (9.62)	1133	–	–	–	–
≥240 mg/m^2^	96 (12.85)	747	1.39 (1.04–1.85)	0.028	1.36 (1.01 to 1.82)	0.039
Epirubicin cumulative dose	142 (6.79)	2092				
<480 mg/m^2^	107 (5.88)	1819	–	–	–	–
≥480 mg/m^2^	35 (12.82)	273	2.35 (1.57–3.53)	<0.001	2.33 (1.55–3.51)	<0.001
BMI CATEGORY	363 (8.66)	4190				
<25 kg/m^2^	146 (7.34)	1988	–	–	–	–
25–30 kg/m^2^	125 (9.23)	1354	1.28 (1.00–1.65)	0.050	1.65 (1.11–2.46)	0.014
>30 kg/m^2^	92 (10.85)	848	1.54 (1.17–2.02)	0.002	2.21 (1.40–3.49)	<0.001

*CI* confidence interval, *N* number, *OR* odds ratio.

**Fig. 3:**
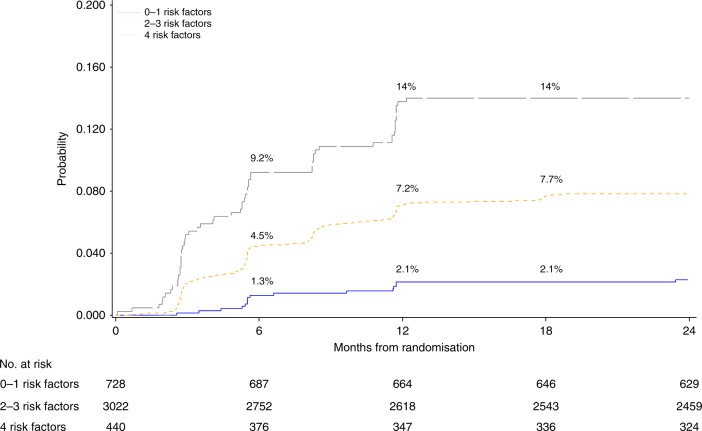
Cumulative incidence of CEs over time, according to number of cardiac risk factors. At 6, 12 and 18 months from randomisation, patients with 0–1 risk factors have an incidence of CE of 1.3%, 2.1% and 2.1%, respectively. Patients with 2–3 risk factors, at the same time-points, have an incidence of CE of 4.5%, 7.2% and 7.7%, respectively. For those with 4 risk factors, the incidences are 9.2%, 14.0% and 14.0%, respectively. Note that any given patient could have accumulated up to 4 risk factors, instead of the 5 identified, since none was exposed both to doxorubicin and epirubicin.

As shown in Fig. 3, CE rates increased from 1.3% to 2.1% at 6 and 12 months of anti-HER2 treatment in the lowest cumulative risk category (0–1 cardiac risk factors). CEs increased from 9.2% to 14% at 6 and 12 months, respectively, in the highest cumulative risk category (4 risk factors).

We further investigated the impact of cardiac risk factors on recovery after a CE. Only the presence of DM was negatively associated with reaching an acute recovery after a CE (*p*-value = 0.031 in Table S6).

## Discussion

In addition to being an oncogenic driver, HER2 is physiologically expressed in the membrane of cardiomyocytes, playing an important role in their homeostasis, particularly under anthracycline treatment.^10^ Upon HER2 pathway inhibition with T, this physiologic function is disturbed, and thus cardiac dysfunction may arise.^11^ This dysfunction demonstrates no ultrastructural changes or myocyte necrosis, unlike what is seen with anthracycline-induced cardiomyopathy.^12^ Yet, in ALTTO, most patients were treated with an anthracycline before HER2 blockade, which might explain why most CEs seems to have occurred in the first 6 months after randomisation: shortly after anthracycline administration, the chances of experiencing a CE with trastuzumab, possibly due to increased oxidative stress, are higher.^13^ Normal signalling via HER2 is critical to the management of oxidative stress: blockade of the anti-oxidative function mediated by neureregulin interaction with HER2, ultimately responsible for the activation of the glutathione reductase system in the heart, facilitates anthracycline-induced accumulation of reactive oxygen species (ROS) and subsequent calcium influx, caspases activation and myocyte necrosis.^14,15^

There are two different hypotheses on the impact of dual HER2 blockade on cardiotoxicity. In the first, it was conjectured that with a more intense HER2 pathway inhibition using two anti-HER2 drugs, more cardiomyocyte dysfunction would occur. Our results, as well as the results from other dual HER2 blockade studies, have not lent support to this hypothesis, though a recent study on T-DM1 cardiotoxicity suggests that more CEs occur with T-DM1+pertuzumab.^3,16–18^ In fact, T + L arm had a lower, albeit non-statistically significant, incidence of CEs. This is in line with the second hypothesis, based on preclinical evidence suggesting a possible cardioprotective mechanism exerted by L: the blockade by T of the HER2 survival pathway in the cardiomyocyte can be compensated with the TKI by means of activation of AMP-activated protein kinases (AMP-K), increasing intracellular ATP thereby protecting against pro-apoptotic stimuli seen during CHF.^19^ Moreover, in an ad-hoc analysis, lapatinib seems to have exerted this compensation mechanism mostly during trastuzumab use, rather than during FU, as the trend for a lower OR for the occurrence of CE was not seen during the FU period. It is important to note, however, that our results cannot be in any way interpreted to suggest lapatinib has a cardioprotective effect, nor that it should be a preferred option for patients with a higher risk of cardiotoxicity, since ALTTO was not designed to test this hypothesis.

After a median follow-up of 6.9 years and 363 CEs, overall CEs rate was generally low (8.6%) and most CEs were asymptomatic (73%). Furthermore, CEs occurred more frequently during anti-HER2 treatment (74%) and were mostly reversible (84%). In the setting of a large, well-conducted randomised trial with strict cardiac monitoring and algorithms for anti-HER2 drug management, these data are reassuring regarding the safety of both T and T + L. Though T + L is not used in the early setting from which these data are generated, it is still a choice for 3rd or further line therapy in advanced disease and therefore the data regarding incidence of CEs and risk factors remains clinically valuable for physicians making treatment and follow-up decisions. Our results are also reassuring about cardiac safety of T used concomitantly with taxanes in the adjuvant setting. Moreover, current guidelines are in line with our data, as it is recommended LVEF monitoring every 3 months during anti-HER2 treatment and once after completion, which fits the timing of occurrence of CE (median of 6.6 months) and the occurrence of some CE during FU (26%) in ALTTO Cardiac.^20^ In fact, future trials could try to address whether it is safe to assess LVEF at wider intervals during anti-HER2 therapy in light of this cardiotoxicity kinetic, especially in patients with none or few cardiac risk factors.

Most patients had LVEF monitored with ECHO rather than MUGA during ALTTO, despite the former disadvantage, in terms of reliance on geometric assumptions and inter-observer variability. Yet, ECHO is widely available in most centres, is more convenient for patients (i.e. less time-consuming), and precludes serial exposure to gamma radiation.^21,22^ Since the method of choice for LVEF measurement was well-balanced between treatment arms, it is therefore safe to assume an even CE assessment between them.

Several aspects of T cardiotoxicity profile from this analysis are in line with the results of an individual-patient level data meta-analysis of HERA, NSBAP B-31 and NCCTG9831, the main trials to test adjuvant T for 1 year. For instance, CEs rates were similar between ALTTO Cardiac and the meta-analysis (9.3% vs 11.3%, respectively), asymptomatic (7.3% in ALTTO Cardiac) and asymptomatic/mildly symptomatic (8.7% in the meta-analysis) CEs corresponded to the majority of events, most events occurred during anti-HER2 treatment (77.6% vs 78.1%) and most patients completed one year of T (84% vs 76.2%).^23^ Nevertheless some, but not all studies reporting real-world data have found a higher rate of CEs with T, although all report that the majority were asymptomatic.^24–27^

So far, five trials testing the non-inferiority for efficacy of shorter adjuvant T regimens compared to the conventional 1-year regimen have been reported. Only 1 of those was able to show non-inferiority of such shorter regimen in terms of disease-free survival, with some criticism towards the statistical assumptions and applicability of its outlier result, especially for patients at higher risk of recurrence.^28–33^ Notwithstanding, physicians facing a treatment duration decision for a patient with a high cardiac risk profile (4 cardiac risk factors) and low tumour burden may opt to pursue the 6-month duration regimen tested in the PERSEPHONE trial, as in that trial and in our study this is associated with less CEs than 12 months of anti-HER2 therapy.

The identified cardiac risk factors are also closely related to what has been identified in the existing literature, except for hypertension, with additional HER2 blockade with L not increasing cardiotoxicity.^34^ Previous trials of dual HER2 blockade with pertuzumab, another clinically available anti-HER2 monoclonal antibody, added to T in the early and metastatic setting also have not shown to increase cardiotoxicity comparted to T alone, possibly because pertuzumab does not share with trastuzumab its autophagy inhibition properties and consequent increment in ROS concentration in cardiomyocytes.^35,36^ Elderly patients (i.e. those older than 65 years), often are underrepresented in clinical trials yet forming a substantial portion of the sample in this sub-analysis (430 patients). In our study, older patients do not seem to be at a greater risk of cardiotoxicity (*p* = 0.064), neither with trastuzumab/lapatinib nor with other modalities of dual HER2 blockade, as previously shown.^37,38^ Therefore, concomitant treatments with two anti-HER2 drugs of otherwise healthy elderly patients may be pursued.

Reassuringly, recovery rates from CE are high, regardless of number of anti-HER2 drugs used and intensity of the event, with more than 80% of patients recovering overall, on average little after 3 months. Nonetheless, we have shown for the first time that, other than a risk factor for cardiotoxicity, DM is also associated with absence of recovery following a CE, thus extra caution may be warranted when treating diabetic patients.

The trade-off between the recurrence risk of a given HER2-positive BC versus the patient’s re-exposure cardiac risk to anti-HER2 treatment following the recovery from an asymptomatic LVEF dysfunction or mildly symptomatic CE must be taken into account: albeit a minority (30%) will develop a second LVEF drop to less than 50%, this incidence seems higher than what is seen for a first CE. Yet, others have shown the feasibility of T in the context of low baseline LVEF, thus further encouraging anti-HER2 treatment despite adverse cardiac scenarios with careful cardiac assessment and optimal adjustment of cardioprotective medications.^39,40^

ALTTO Cardiac has several strengths, namely the large population with long follow-up submitted to rigorous cardiac monitoring, including a second LVEF assessment in a shorter interval following an asymptomatic or mildly symptomatic CE, in order to confirm the CE and decrease the rate of false-positive ultra-sound readings, and with reported CEs being managed with the aid of a cardiac advisory board, yet some weaknesses must be recognised.^41^ First, ALTTO was not powered to show a true statistical difference on CE rates between its treatment arms, thus we cannot completely rule out that the addition of L to T does not produces an increment on CE rates compared to T alone. Second, rigorous selection and monitoring of patients performed often does not mirror that of those treated outside clinical trials, meaning the latter may be at a higher risk of cardiotoxicity than in this analysis due to older age and higher number of comorbidities. Third, the absence of a dynamic cardiac biomarkers assessment, such as high-sensitivity troponin I or B-natriuretic peptide, and global longitudinal strain assessment during anti-HER2 treatment, though their true impact on anti-HER2 treatment management is yet to be defined.^20^ The ALTTO’s dataset also precluded further analysis on the impact of cardioprotective medications commencement, upon the development of a CE, on cardiac recovery and recurrence rates. Lastly, analysis regarding smoking status, an established cardiac risk factor, could not be performed, as this variable was not collected.^42^

## Conclusion

Dual HER2 blockade with T + L is a safe regimen from a cardiac standpoint. Most CEs are asymptomatic, occurs during anti-HER2 treatment and further accumulate from the 6th to the 12th month of treatment, thus cardiac monitoring in this period should be followed according to guidelines, especially for patients with one or more of the following risk factors: pre-treatment LVEF < 55%, BMI > 30 kg/m^2^, high cumulative anthracycline exposure and DM. Future trials could address whether a low cardiac risk population could be safely monitored with a less intense LVEF schedule of assessment, thus helping to reduce financial costs associated with anti-HER2 treatment.

## Supplementary information

Supplementary material

## Data Availability

The authors declare that all data supporting the findings of this study are available within the article and its Supplementary Information files or are available from the corresponding author upon reasonable request.
