# Supplementary material for: Long-term cardiac outcomes of patients with HER2-positive breast cancer treated in the adjuvant lapatinib and/or trastuzumab Treatment Optimization Trial
Source: Br J Cancer. 2020 Mar 16;122(10):1453–60. doi: 10.1038/s41416-020-0786-x (PMC7217956; doi:10.1038/s41416-020-0786-x)
Supplement: Supplementary file 1 — Supplementary material [file 41416_2020_786_MOESM1_ESM.docx]

ALTTO Cardiac – Supplementary material

Table S1 - cardiac endpoints definitions according to ALTTO protocol version 12, released in July 2016 versus ALTTO Cardiac definitions:

|  | **ALTTO protocol v10** | **ALTTO Cardiac** |
| --- | --- | --- |
| **Primary (cardiac) endpoint** | Incidence of cardiac death + severe symptomatic HF | Incidence of any CE (summed incidence of asymptomatic CE + symptomatic CE + cardiac death) |
| **Secondary (cardiac) endpoints** | Incidence of class I + II HF of NYHA | Incidence of asymptomatic CE; Incidence of symptomatic CE;  Incidence of cardiac death;  Risk factor(s) for occurrence of any CE (cardiac risk factors);  Time to development of CE;  Incidence and time to recovery from CE. |
| **Classification according to NYHA class** | Asymptomatic or mildly symptomatic HF = class I or II;  Severe symptomatic HF = class III + IV | Asymptomatic CE = asymptomatic Left Ventricular dysfunction (class I)  Symptomatic CE = CHF class II + III + IV |

Table S2 – All population characteristics, according to treatment arm:

| **Baseline Features** | **T (N = 2097) N (%)** | **T+L (N = 2093) N (%)** | **p-value** |
| --- | --- | --- | --- |
| Age (year) categorized |  |  | 0.918 |
| <35 | 113 (5) | 126 (6) |  |
| 35-39 | 173 (8) | 177 (8) |  |
| 40-49 | 638 (30) | 623 (30) |  |
| 50-64 | 957 (46) | 953 (46) |  |
| ≥65 | 216 (10) | 214 (10) |  |
| Menopausal status at randomization |  |  | 0.932 |
| Premenopausal | 908 (43) | 909 (43) |  |
| Postmenopausal | 1189 (57) | 1184 (57) |  |
| Body Mass Index (BMI) |  |  | 0.916 |
| <25 | 999 (48) | 989 (47) |  |
| 25-30 | 679 (32) | 675 (32) |  |
| >30 | 419 (20) | 429 (20) |  |
| Ethnicity |  |  | 0.424 |
| Asian | 555 (26) | 546 (26) |  |
| Black | 25 (1) | 38 (2) |  |
| White | 1451 (69) | 1445 (69) |  |
| Other/Missing | 66 (3) | 64 (3) |  |
| Co-morbidity |  |  | 0.470 |
| Yes | 588 (28) | 566 (27) |  |
| No | 1509 (72) | 1527 (73) |  |
| Baseline LVEF |  |  | 0.765 |
| 50-54% | 98 (5) | 102 (5) |  |
| > 64% | 934 (45) | 937 (45) |  |
| 55% - 64% | 1065 (51) | 1053 (50) |  |
| Missing | 0 | 1 (<1) |  |
| LVEF evaluation method |  |  | 0.616 |
| ECHO | 1588 (76) | 1571 (75) |  |
| MUGA | 509 (24) | 522 (25) |  |
| Hypertension |  |  | 0.293 |
| Yes | 471 (22) | 442 (21) |  |
| No | 1626 (78) | 1651 (79) |  |
| Diabetes Mellitus |  |  | **0.024** |
| Yes | 128 (6) | 95 (5) |  |
| No | 1969 (94) | 1998 (95) |  |
| Hypercholesterolemia |  |  | 0.290 |
| Yes | 179 (9) | 160 (8) |  |
| No | 1918 (91) | 1933 (92) |  |
| Pathological tumor size (cm) |  |  | 0.964 |
| No applicable (neo-adjuvant chemotherapy) | 181 (9) | 168 (8) |  |
| 0 - <2 | 701 (33) | 708 (34) |  |
| ≥2 - <5 | 1053 (50) | 1054 (50) |  |
| ≥5 | 153 (7) | 155 (7) |  |
| Unknown | 9 (<1) | 8 (<1) |  |
| Lymph node status |  |  | 0.878 |
| No applicable (neo-adjuvant chemotherapy) | 181 (9) | 168 (8) |  |
| Negative | 844 (40) | 845 (40) |  |
| One to three positive nodes | 603 (29) | 617 (29) |  |
| Four or more positive nodes | 469 (22) | 463 (22) |  |
| Histologic grade |  |  | 0.181 |
| Well differentiated | 48 (2) | 51 (2) |  |
| Moderately differentiated | 744 (35) | 774 (37) |  |
| Poorly differentiated | 1237 (59) | 1179 (56) |  |
| Not assessed/Unknown | 68 (3) | 89 (4) |  |
| Hormone receptor status |  |  | 0.869 |
| Negative | 897 (43) | 890 (43) |  |
| Positive | 1200 (57) | 1203 (57) |  |
| Type of primary surgery |  |  | 0.606 |
| Mastectomy | 1166 (56) | 1164 (56) |  |
| Breast-conserving procedure | 931 (44) | 928 (44) |  |
| Missing/Unknown | 0 | 1 (<1) |  |
| Radiotherapy |  |  | 0.816 |
| Yes | 1486 (71) | 1490 (71) |  |
| No | 611 (29) | 603 (29) |  |
| Radiotherapy laterality* |  |  | 0.165 |
| Left | 745 (50) | 790 (53) |  |
| Right | 736 (50) | 698 (47) |  |
| Bilateral | 5 (<1) | 2 (<1) |  |
| Timing of chemotherapy |  |  | 0.752 |
| Sequential | 1147 (55) | 1155 (55) |  |
| Concurrent | 950 (45) | 938 (45) |  |
| Chemotherapy regimen |  |  | 0.902 |
| Anthracycline followed by taxane | 1985 (95) | 1983 (95) |  |
| Non-Anthracycline (docetaxel+carboplatin) | 112 (5) | 110 (5) |  |
| Median doxorubicin cumulative dose | 237.62 (mg/m2) | 237.84 (mg/m2) | - |
| Median epirubicin cumulative dose | 350.86 (mg/m2) | 349.75 (mg/m2) | - |
| Median follow-up in years (IQR) | 6.92 (6.00-7.13) | 6.93 (6.00-7.14) | - |
| Median follow-up in months (IQR) | 83.09 (72.05-85.5) | 83.19 (72.02-85.68) | - |

* Percentages derived using patients treated with radiotherapy as denominator

Legends: L = lapatinib; LVEF = left ventricular ejection fraction; T = trastuzumab

Table S3 – Summary of treatment discontinuation

|  | **T (N = 2097) N (%)** | **T + L (N = 2093) N (%)** |
| --- | --- | --- |
| Trastuzumab completion status |  |  |
| Completed trastuzumab | 1753 (84) | 1708 (82) |
| Discontinued trastuzumab | 344 (16) | 385 (18) |
| Reasons for trastuzumab discontinuation* |  |  |
| Safety | 123 (36) | 135 (35) |
| Recurrence of disease | 51 (15) | 26 (7) |
| Other reasons | 170 (49) | 224 (58) |
| Safety reasons for trastuzumab discontinuation** |  |  |
| Cardiac safety | 86 (70) | 78 (58) |
| Other safety | 37 (30) | 57 (42) |
| Lapatinib completion status |  |  |
| Completed lapatinib | NA | 1419 (68) |
| Discontinued lapatinib |  | 674 (32) |
| Reasons for lapatinib discontinuation* |  |  |
| Safety | NA | 405 (60) |
| Recurrence of disease |  | 23 (3) |
| Other reasons |  | 246 (36) |
| Safety reasons for lapatinib discontinuation** |  |  |
| Cardiac safety | NA | 74 (18) |
| Other safety |  | 331 (82) |

* Discontinued trastuzumab/lapatinib used as denominator to derive percentages

** Safety used as denominator to derive percentages

Legends: NA = not available.

Table S4 – Summary of acute recovery and second LVEF drop

|  | **T+L** | **Median* (months)** | **Range** (months)** | **T** | **Median* (months)** | **Range** (months)** |
| --- | --- | --- | --- | --- | --- | --- |
| **Any CE** | 164 |  |  | 195 |  |  |
| Reached acute recovery*** | 138(84.1%) |  |  | 163(83.6%) |  |  |
| Time to acute recovery |  | 3.5 | ( 0.1 - 23.9) |  | 3.3 | ( 0.0 - 79.0) |
| Occurrence of LVEF drop < 50% after acute recovery | 43(31.2%) |  |  | 47(28.8%) |  |  |
| Re-exposure to anti-HER2 treatment after acute recovery | 32 (23.2%) |  |  | 58 (35.6%) |  |  |
| Occurrence of LVEF drop < 50% after acute recovery and re-exposure to anti-HER2 therapy | 12 (37.5%) |  |  | 15 (25.9%) |  |  |
| No re-exposure to anti-HER2 treatment after acute recovery | 106 (76.8%) |  |  | 105 (64.4%) |  |  |
| Occurrence of LVEF drop < 50% after acute recovery without re-exposure to anti-HER2 therapy | 31 (29.2%) |  |  | 32 (30.5%) |  |  |
| **Asymptomatic CE** | 110 |  |  | 155 |  |  |
| Reached acute recovery | 92(83.6%) |  |  | 128(82.6%) |  |  |
| Time to acute recovery |  | 2.9 | ( 0.1 - 23.9) |  | 3.1 | ( 0.0 - 37.8) |
| Occurrence of LVEF drop < 50% after acute recovery | 27(29.3%) |  |  | 32(25.0%) |  |  |
| **Symptomatic CE** | 54 |  |  | 40 |  |  |
| Reached acute recovery | 46(85.2%) |  |  | 35(87.5%) |  |  |
| Time to acute recovery |  | 4.2 | ( 0.1 - 21.9) |  | 5.6 | ( 0.5 - 79.0) |
| Occurrence of LVEF drop < 50% after acute recovery | 16(34.8%) |  |  | 15(42.9%) |  |  |

* Kaplan-Meier estimate of median

** Range excluding censored values

***4 cardiac deaths events were excluded to derive denominators

Table S5 – Summary of uni and multivariate analysis of baseline characteristics tested to predict CEs

| Baseline Characteristic | Cardiac Events (%) | N | Univariate OR (95%CI) | Univariate P-value | Multivariate OR (95%CI) | Multivariate P-value |
| --- | --- | --- | --- | --- | --- | --- |
| Pre anti-HER2 LVEF | 363 (8.67) | 4189 |  |  |  |  |
| > 64% | 102 (4.82) | 2118 | - | - | - | - |
| 55% - 64% | 225 (12.03) | 1871 | 2.70 (2.12 to 3.44) | **<0.001** | 2.32 (1.61 to 3.35) | **<0.001** |
| < 55% | 36 (18.00) | 200 | 4.34 (2.87 to 6.55) | **<0.001** | 3.10 (1.54 to 6.25) | **0.002** |
| Diabetes Mellitus | 363 (8.66) | 4190 |  |  |  |  |
| No | 330 (8.32) | 3967 | - | - | - | - |
| Yes | 33 (14.80) | 223 | 1.91 (1.30 to 2.82) | **<0.001** | 1.85 (1.25 to 2.75) | **0.002** |
| Anthracycline use | 363 (8.66) | 4190 |  |  |  |  |
| No | 16 (7.21) | 222 | - | - | - | - |
| Yes | 347 (8.74) | 3968 | 1.23 (0.73 to 2.08) | 0.429 | 1.68 (0.18 to 15.58) | 0.648 |
| Doxorubicin cumulative dose | 205 (10.90) | 1880 |  |  |  |  |
| < 240mg/m2 | 109 (9.62) | 1133 | - | - | - | - |
| ≥240mg/m2 | 96 (12.85) | 747 | 1.39 (1.04 to 1.85) | **0.028** | 1.36 (1.01 to 1.82) | **0.039** |
| Epirubicin cumulative dose | 142 (6.79) | 2092 |  |  |  |  |
| < 480mg/m2 | 107 (5.88) | 1819 | - | - | - | - |
| ≥480mg/m2 | 35 (12.82) | 273 | 2.35 (1.57 to 3.53) | **<0.001** | 2.33 (1.55 to 3.51) | **<0.001** |
| BMI CATEGORY | 363 (8.66) | 4190 |  |  |  |  |
| <25 | 146 (7.34) | 1988 | - | - | - | - |
| 25-30 | 125 (9.23) | 1354 | 1.28 (1.00 to 1.65) | **0.050** | 1.65 (1.11 to 2.46) | **0.014** |
| >30 | 92 (10.85) | 848 | 1.54 (1.17 to 2.02) | **0.002** | 2.21 (1.40 to 3.49) | **<0.001** |
| Age dichotomized at 65 | 363 (8.66) | 4190 |  |  |  |  |
| <65 | 314 (8.35) | 3760 | - | - | - | - |
| ≥65 | 49 (11.40) | 430 | 1.41 (1.03 to 1.94) | **0.034** | 1.36 (0.98 to 1.88) | 0.064 |
| Hypertension | 363 (8.66) | 4190 |  |  |  |  |
| No | 279 (8.51) | 3277 | - | - | - | - |
| Yes | 84 (9.20) | 913 | 1.09 (0.84 to 1.41) | 0.514 | 0.89 (0.67 to 1.17) | 0.402 |
| Hypercholesterolemia | 363 (8.66) | 4190 |  |  |  |  |
| No | 334 (8.67) | 3851 | - | - | - | - |
| Yes | 29 (8.55) | 339 | 0.99 (0.66 to 1.46) | 0.941 | 0.90 (0.60 to 1.36) | 0.629 |
| Radiotherapy | 363 (8.66) | 4190 |  |  |  |  |
| No | 111 (9.14) | 1214 | - | - | - | - |
| Yes | 252 (8.47) | 2976 | 0.92 (0.73 to 1.16) | 0.481 | 0.96 (0.75 to 1.21) | 0.709 |
| Radiotherapy laterality | 252 (8.49) | 2969 |  |  |  |  |
| Right | 127 (8.86) | 1434 | - | - | - | - |
| Left | 125 (8.14) | 1535 | 0.91 (0.70 to 1.18) | 0.486 | 0.92 (0.71 to 1.19) | 0.509 |
| Anti-HER2 arm | 363 (8.66) | 4190 |  |  |  |  |
| T | 197 (9.39) | 2097 | - | - | - | - |
| T + L | 166 (7.93) | 2093 | 0.83 (0.67 to 1.03) | 0.093 | 0.85 (0.68 to 1.05) | 0.139 |
| Anti-HER2 arm (during anti-HER2 therapy) | 270 (6.44) | 4190 |  |  |  |  |
| T | 153 (7.30) | 2097 | - | - | - | - |
| T + L | 117 (5.59) | 2093 | 0.75 (0.59 to 0.96) | **0.025** | 0.77 (0.60 to 0.99) | **0.038** |
| Anti-HER2 arm (during FU) | 93 (2.22) | 4190 |  |  |  |  |
| T | 44 (2.10) | 2097 | - | - | - | - |
| T + L | 49 (2.34) | 2093 | 1.12 (0.74 to 1.69) | 0.594 | 1.14 (0.76 to 1.73) | 0.525 |

Table S6 – association of cardiac risk factors with acute recovery and absence of acute-recovery following a CE

| **Cardiac Risk Factor** | **N of Recovery (%)** | **N of No-Recovery (%)** | **p-value** |
| --- | --- | --- | --- |
| **Baseline LVEF** |  |  | 0.131 |
| 50-54% | 31 (88.6) | 4 (11.4) | - |
| 55% - 64% | 89 (89.0) | 11 (11.0) | - |
| > 64% | 181 (80.8) | 43 (19.2) | - |
| **Presence of DM** |  |  | **0.031** |
| Yes | 21 (70.0) | 9 (30.0) | - |
| No | 280 (82.1) | 49 (14.9) | - |
| **Doxorubicin** |  |  | 0.570 |
| < 240mg/m2 | 92 (86.0) | 15 (14.0) | - |
| ≥ 240mg/m2 | 81 (85.3) | 14 (14.7) | - |
| NA | 128 (81.5) | 29 (18.5) | - |
| **Epirubicin** |  |  | 0.938 |
| < 480mg/m2 | 88 (83.0) | 18 (17.0) | - |
| ≥ 480mg/m2 | 29 (82.9) | 6 (17.1) | - |
| NA | 184 (84.4) | 34 (15.6) | - |
| **BMI** |  |  | 0.131 |
| <25 | 107 (86.3) | 17 (13.7) | - |
| 25-30 | 123 (86.0) | 20 (14.0) | - |
| >30 | 71 (77.2) | 21 (22.8) | - |
|  | | | |
| **Total** | 301 (83.8) | 58 (16.2) | - |


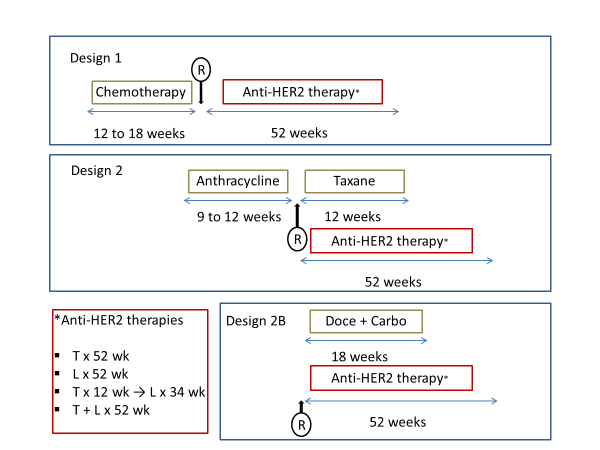


Fig. S1 – Modalities of anti-HER2 therapy, according to randomization arm, and chemotherapy administration, according to physician`s choice.

Legends: Carbo = carboplatin; Doce = docetaxel; L = lapatinib; R = randomization; T = trastuzumab; wk = weeks.


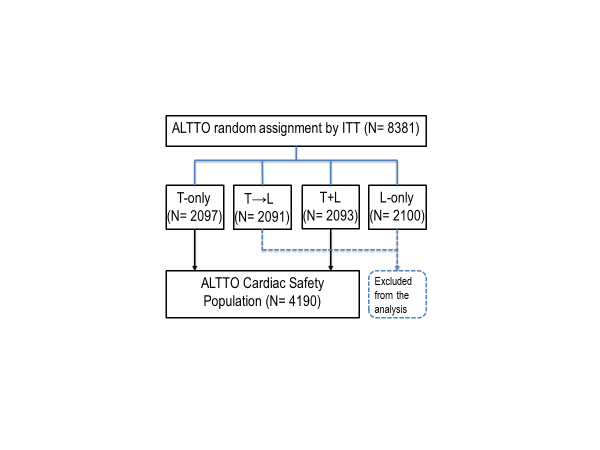


Fig. S2 – ALTTO Cardiac Consort diagram

Legends: ITT = intention-to-treat; L = lapatinib; T = trastuzumab.


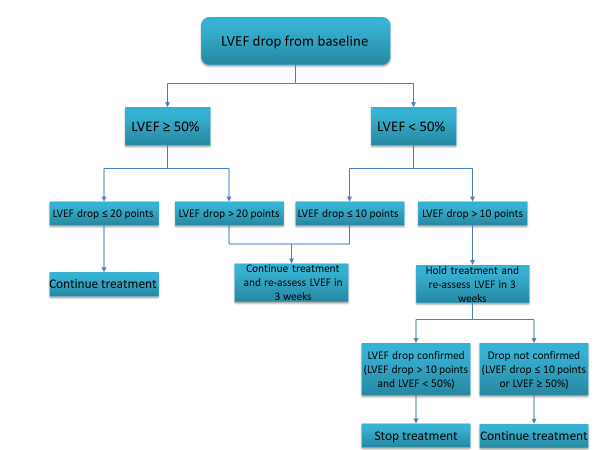


Fig. S3 – Algorithm for anti-HER2 treatment management on the event of heart failure NYHA class I and II

Legend: LVEF = left ventricular ejection fraction.
